# California State Veterinary Medical Association

**Published:** 1892-12

**Authors:** R. C. Archibald

**Affiliations:** Secretary


*California State Veterinary Medical Association.*—A meeting of the California State Veterinary Medical Association was held in the Baldwin Hotel, San Francisco, September 14, 1892.

The meeting was called to order by the Vice-President, Dr. W. F. Egan, of San Francisco. Upon roll-call the following gentlemen responded: Drs. Egan, Witherspoon, Pierce, Masoero, Spencer, Sr., Spencer, Jr., Fox, Burns, Orvis, Wadams Powers, Neif and Archibald. Visitors: Drs Harry and Thomas Carpenter. Letters of regret were then read from absent members.

Dr. Pierce, of Oakland, then read a very practical and instructive paper on "Fractures." The essayist described numerous cases which had come under his notice. He also described his methods of treating same.

A lively and animated discussion followed, in which all the members present joined.

The Committee on Legislature were then called upon for their report. The Secretary read the report, and also read the views of the Southern members on the subject. Drs. H. and T. Carpenter were asked to give their opinion. Thereupon Dr. T. Carpenter rose and gave a very exhaustive speech, reviewing the subject both from a professional and unprofessional standpoint.

After a long discussion, it was finally decided that a meeting of the veterinary practitioners of California, irrespective of association, be called, and the subject-matter of Legislature be laid before them. It was also decided that the Association should adjourn for a short time, so that some plans could be made to bring about such a meeting

A motion to adjourn was carried.

The meeting being again called to order, the following-named gentlemen were elected members, viz: Dr. J. C. C Price, Los Angeles; Dr. R. H. Powers, Chico, and Dr. F. W. Skaife, San Francisco. The Secretary then proposed the names of the following gentlemen: Dr. J. Graham, Fresno; Dr. Carmichael, Hanford; Dr. Rowland Lord, San Jose.

The Secretary then spoke at some length on the subject of having as many of the members as possible join the United States Veterinary Medical Association, and having the California State Veterinary Medical Association well represented at the meeting of the above association in Chicago next year. He endeavored to portray the benefits the veterinary profession of the United States would receive from this association. He stated that it was his opinion that the veterinary practitioner would receive more benefits from this organization than from his local association.

The Chair then declared nominations for officers for the ensuing year in order. The following gentlemen were nominated for officers: President, Dr. W. F. Egan; Vice-President, Drs. Spencer, Jr., and Whittlesey; Secretary, Dr. R. A. Archibald; Treasurer, Dr. C. B Orvis; Directors, Drs. Spencer, Sr., Burns and Wadams; Board of Examiners, Drs. Maclay, Masoero, Orvis, Rowland and Morrison.

There being no further business before the meeting, it adjourned to meet in Los Angeles December 14, 1892 R. C. ARCHIBALD, D.V.S., *Secretary.*

